# Intestinal Bacteria

**Published:** 1903-01-10

**Authors:** 


### INTESTINAL BACTERIA.

Some interesting observations have lately been made by Dr. Lorrain Smith [1] and Dr. Tennant in regard to the growth of bacteria in the intestine. It has for some time been well known, they say, that if cultivations be made of the bacterial contents present in different parts of the intestine the colonies will be found to differ according to the part investigated. Speaking generally, bacteria are much more abundant in the large than in the small intestines. But the intestines have the power of regulating the kind of bacteria which shall survive as well as their number, the universal presence of bacilli belonging to the coli communis and proteus group, showing that the conditions maintained in the normal intestine permit of the survival of these bacteria, while numbers of other species ingested with the food are more or less destroyed. It is important to note that this power of regulating bacterial growth is destroyed or modified in a number of diseases, especially in those which originate primarily in the intestine. Thus non-specific irritations of various kinds, such as the disturbance caused by worms or by conditions of catarrh, may so interfere with the controlling power of the intestines as to cause, or to allow, an abundant bacterial growth to take place in areas in which such growth is normally present in only a limited degree. In this way we are naturally led to the question how far the morbid processes which go on in those diseases which are due to some special microbe, as, for example, the typhoid bacillus, are due directly to the specific irritation caused by that micro-organism, or how far they may be due to an abnormal growth of ordinary bacteria. The results of the investigation show that one of the first places in which a disturbance of the regulation of bacterial growth takes place is in the region of the ileo-cæcal junction, the bacteria which normally are present in the large intestine not only becoming more abundant in their usual position, but encroaching upon areas in which they usually are not to be found, such as the region of the ileo-cæcal valve and, in less degree, the small intestine. We are led then to believe on the one hand that preceding conditions of irritation may, by diminishing intestinal control, have the effect of permitting the growth of such organisms as are responsible for the production of appendicitis or typhlitis, of typhoid fever, and of intestinal tuberculosis, all of which tend to locate themselves about the area above referred to. On the other hand we see that the existence of diseases affecting this area, whether caused by specific organisms or by mere "irritation," would in turn allow of a great increase in the growth of those micro-organisms which although normal to other parts of the intestine are not normal to this area.

It is possible then that the symptom complex which we see in certain diseases may not be entirely due to the original pathogenic organism (for example the bacillus typhosus), but also in large degree to the overgrowth of bacillus coli which has resulted from loss of control over bacterial growth, arising from the morbid condition of the mucous membrane. So do the different "causes" of disease act and react upon one another.

[1] Brit. Med. Jour., Dec. 27.

